# Interactions between gut microbiota and berberine, a necessary procedure to understand the mechanisms of berberine

**DOI:** 10.1016/j.jpha.2021.10.003

**Published:** 2021-10-21

**Authors:** Hao Cheng, Juan Liu, Yuzhu Tan, Wuwen Feng, Cheng Peng

**Affiliations:** State Key Laboratory of Southwestern Chinese Medicine Resources, School of Pharmacy, Chengdu University of Traditional Chinese Medicine, Chengdu, 611137, China

**Keywords:** Berberine, Gut microbiota, Oral bioavailability, Traditional Chinese medicines, Short chain fatty acids

## Abstract

Berberine (BBR), an isoquinoline alkaloid, has been found in many plants, such as *Coptis chinensis* Franch and *Phellodendron chinense* Schneid. Although BBR has a wide spectrum of pharmacological effects, its oral bioavailability is extremely low. In recent years, gut microbiota has emerged as a cynosure to understand the mechanisms of action of herbal compounds. Numerous studies have demonstrated that due to its low bioavailability, BBR can interact with the gut microbiota, thereby exhibiting altered pharmacological effects. However, no systematic and comprehensive review has summarized these interactions and their corresponding influences on pharmacological effects. Here, we describe the direct interactive relationships between BBR and gut microbiota, including regulation of gut microbiota composition and metabolism by BBR and metabolization of BBR by gut microbiota. In addition, the complex interactions between gut microbiota and BBR as well as the side effects and personalized use of BBR are discussed. Furthermore, we provide our viewpoint on future research directions regarding BBR and gut microbiota. This review not only helps to explain the mechanisms underlying BBR activity but also provides support for the rational use of BBR in clinical practice.

## Highlights

• Low bioavailability enables interactions between berberine and the gut microbiota.
• Berberine can shape the composition and metabolism of the gut microbiota.
• Gut microbiota can metabolize and transform berberine.
• Personalized use of berberine can reduce the occurrence of side effects.

## Graphical abstract

**Figure:**
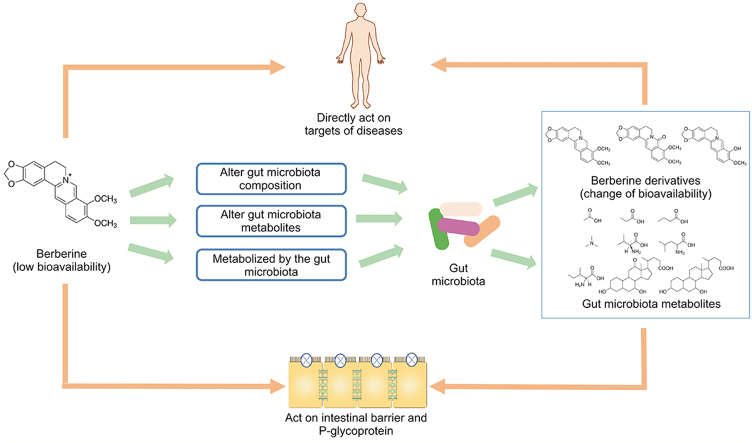

## 1 Introduction

Berberine (BBR) is alkaloid that belongs to isoquinoline group and has a molecular formula of C_20_H_18_NO_4_. BBR can be extracted from various plant families, including *Annonaceae*, *Berberidaceae*, *Menispermaceae*, and *Papaveraceae* [1]. Over the past decades, BBR has emerged as an important compound in traditional Chinese medicine because of its wide range of applications [2,3]. Numerous studies have demonstrated that BBR exhibits positive pharmacological effects in many diseases such as cancer [4,5], inflammation [6], bacterial infection [7], reperfusion injury [8], and non-alcoholic fatty liver (NAFL) disease [9]. Therefore, it has a great clinical value. In contrast to its strong pharmacological effects, the oral bioavailability of BBR is extremely low [10]. The absolute bioavailability and peak concentration (*c*_max_) of orally administered 100 mg/kg BBR in rats were 0.68% and 9.48 ± 3.40 ng/mL, respectively [11]. The *c*_max_ value of 50 mg/kg BBR in dogs was 36.88 ± 23.45 ng/mL [12]. The contradiction between the low oral bioavailability of BBR and strong pharmacological actions of BBR has perplexed pharmacologists for a long time [13].

A large number of microorganisms are distributed in almost all parts of the body, such as the mouth, gastrointestinal tract, and vagina [14]. Gut microbiota is a group of microbes that mainly inhabit the intestine and account for over 70% of human microorganisms. Most of the gut microbiota are bacteria, mainly belonging to four phyla: *Bacteroidetes*, *Actinobacteria*, *Firmicutes*, and *Proteobacteria* [15]. Normal composition and metabolism of gut microbiota is vital for the maintenance of health, and disturbances in the gut microbiota can lead to the occurrence of various diseases, such as diabetes [16], diarrhea [17], and colitis [18]. In contrast, restoration of gut microbiota by various methods such as administration of antibiotics and probiotics and fecal microbiota transplantation can ameliorate diseases [19,20]. Besides the direct roles of gut microbiota in disease development and treatment, many researches have demonstrated that gut microbiota can interact with drugs in the past two decades [21]. On the one hand, gut microbiota can metabolize drugs into products with different potencies; on the other hand, drugs can influence gut microbiota composition and its metabolism [22,23]. Because these interactions can significantly influence the efficacy and toxicity of drugs, it has become essential to study the mechanisms of drugs with respect to the gut microbiota [24].

Due to the extremely low bioavailability of BBR, BBR can easily reach the intestines and interact with the gut microbiota. These direct interactions include modulation of composition and metabolism of gut microbiota by BBR and transformation of BBR by gut microbiota [25,26]. Gut microbiota has the capability to synthesize an array of metabolites essential for maintaining normal body functions, such as trimethylamine (TMA), short-chain fatty acids (SCFAs), bile acids (BAs), and branched chain amino acids (BCAAs) [27,28]. BBR can regulate these metabolites by regulating the amounts of gut bacteria that produce these metabolites and the expression or activity of enzymes responsible for the production of these metabolites [29,30]. In addition, BBR can be metabolized in the intestinal tract by gut microbiota through processes including oxidation, reduction, and demethylation, thereby forming products with different properties [31]. The pharmacological activity of BBR can be significantly influenced by these interactions. Although one review has discussed the influence of BBR on gut microbiota composition [25], no systemic summary or discussion about the interactive relationship between gut microbiota and BBR has been reported. In this review, we summarize the interactions between gut microbiota and BBR and the influences of these interactions on pharmacological effects, hoping to provide an explanation for the contradiction between low bioavailability and strong pharmacological effects of BBR, and to rationally apply BBR in clinical practice.

## 2 BBR directly regulates gut microbiota

In the gastrointestinal tract, the normal composition of gut microbiota can restrain the invasion of pathogens by inducing host release of antimicrobial materials, competitive consumption of nutrient sources, and occupation of attachment sites [32]. In addition, gut microbiota can generate and release a battery of metabolites such as BCAAs and trimethylamine *N*-oxide (TMAO), which are favorable or unfavorable to hosts depending on context. Therefore, regulating gut microbiota composition and metabolism is regarded as an important approach to treating diseases [33]. Because of its low bioavailability, BBR can influence the gut microbiota composition and metabolism by directly interacting with gut microbiota, thus assisting in the amelioration of diseases.

### 2.1 BBR regulates the composition of gut microbiota

BBR is a natural compound with direct antibacterial effects [34]. BBR (78 μg/mL) has a direct antibacterial effect on *Streptococcus agalactiae*, and the antibacterial effect increases as BBR concentration and exposure time increase [35]. BBR (32 μg/mL) can inhibit the growth of *Candida albicans* by inhibiting biofilm formation [36], and 128 μg/mL BBR can inhibit methicillin-resistant *Staphylococcus aureus* (*S. aureus*) [37]. When *Escherichia coli* (*E. coli*) is exposed to BBR (750 μg/mL), the cellular lifespan of *E*. *coli* decreases with prolonged exposure time [38]. In addition, BBR has direct inhibitory effects on bacteria such as *Clostridium difficile*, *Actinobacillus*, and *Bacillus subtilis* [[39], [40], [41]]. These studies indicate the direct antibacterial activity of BBR.

Due to its low oral availability and direct antibacterial effect, BBR can easily reach the colon and change the gut microbiota composition. Some researchers have revealed that BBR can regulate gut microbiota composition in normal animals (Table 1) [[29], [42], [43], [44], [45], [46], [47], [48], [49], [50], [51], [52], [53], [54], [55], [56], [57], [58], [59], [60], [61], [62], [63], [64], [65], [66], [67], [68], [69], [70]]. Pan et al. [42] studied the influence of BBR on the gut microbiota composition of juvenile grass carp and observed the increased abundances of *Bacteroidetes*, *Proteobacteria*, and *Firmicutes* and the significantly decreased abundance of *Fusobacteria*. In normal mice, BBR can decrease the levels of *Clostridium cluster* (*C. cluster*) XIVa and *C*. *cluster* IV in the intestines [29]. In another study, a decrease in *Ruminococcus gnavus* and *Ruminococcus schinkii* and an increase in *Bacteroides* were observed in normal mice after treatment with BBR [43].

**Table 1:** The changes in gut microbiota composition caused by BBR.

Animal Models	Dosage of BBR	Key findings	Refs.
Normal C57BL/6 wild type mice	100 mg/kg daily for one week	*Clostridiumcluster* XIVa and *Clostridiumcluster* IV were decreased; the proportion of *Firmicutes*/*Bacteriodetes* was decreased.	[29]
Normal juvenile grass carp	30 mg/kg daily for 7 days	The relative abundances of *Bacteroides* and *Proteus* were increased; *Fusobacteria* was decreased.	[42]
Seven-week-old male C57BL/6 mice	100 mg/kg daily for two weeks	*Bacteroides* was increased; *Lactobacillus acidophilus*, *Lactobacillus murinus*, *Lactococcus lactis*, *Ruminococcusgnavus*, and *Ruminococcusschinkii* were decreased.	[43]
HFD-induced insulin resistance rats	200 mg/kg daily for eight weeks	*Bifidobacterium and Lactobacillus* were enriched; *Escherichiacoli* was inhibited.	[44]
HFD-induced atherosclerosis male *ApoE*^-/-^ mice	0.5 g/L in drinking water for 14 weeks.	The diversity of intestinal microbial community was decreased; *Verrucomicrobia*, *Akkermansia,* and *Bacteroides* were increased.	[45]
HFD-induced atherosclerosis male *ApoE*^-/-^ mice	50 mg/kg twice weekly for 12 weeks	*Firmicutes* and *Verrucomicrobia* were enriched; *Bacteroidetes* and *Proteobacteria* were decreased.	[46]
HFD-induced atherosclerosis in mice	100 mg/kg daily for 13 weeks	*Proteobacteria* was reduced; *Actinobacteria*, *Blautia, Roseburia*, *Blautia*, *Allobaculum*, *Alistipes*, and *Turicibacter* were enriched.	[47]
HFD-induced NAFL in rats	150 mg/kg daily for 4 weeks	*Bacteroidetes* and *Proteobacteria* were significantly increased; *Firmicutes* and *Cyanobacteria* were significantly decreased.	[48]
HFD-induced NAFL disease in rats	150 mg/kg daily for 4 weeks	*Faecalibacterium prausnitzii* was reduced; *Bacteroides* was elevated.	[49]
DSS-induced ulcerative colitis in rats	100 mg/kg daily for 6 days	*Mucispirillum schaedleri* and *Bacteroides uniformis* were decreased; *Lachnospiraceae bacterium*, *Faecalibaculum rodentium*, *Corynebacterium glutamicum*, *Akkermansia muciniphila*, *Ruminococcus flavefaciens*, and *Bifidobacterium pseudolongum* were increased.	[50]
DSS-induced ulcerative colitis in rats	40 mg/kg daily for 10 days	*Desulfovibrio* was decreased; *Eubacterium* and *Bacteroides* were increased.	[51]
Colorectal cancer mice induced by azoxymethane/DSS	100 mg/kg daily for 10 weeks	*Actinobacteria*, *Verrucomicrobia*, *Bifidobacterium*, *Barnesiella*, and *Odoribacter* were decreased; *Alloprevotella*, *Flavonifractor*, *Oscillibacter*, and *Parabacteroides* were increased.	[52]
ob/ob mice	100 mg/kg daily for 10 days	*Enterobacter*, *Escherichia-Shigella, Incertae sedis*, *Akkermansia*, and *Bacteroides were* increased.	[53]
db/db mice with T2D	100 mg/kg daily for 55 days	*Saccharibacteria*, *Deferribacteres*, *Actinobacteria*, and *Firmicutes* were reduced; *Verrucomicrobia* was increased.	[54]
db/db mice with T2D	136.5 mg/kg daily for 11 weeks	*Butyricimonas*, *Lactobacillus*, *Coprococcus*, *Ruminococcus*, and *Akkermansia* were increased; *Prevotella* and *Proteuswere* were reduced.	[55]
Collagen induced arthritis in rats	200 mg/kg daily for 14 days	*Blautia, Butyricicoccus, and Parabacteroides were increased; Prevotella, Paraprevotella, and Coprococcus were decreased.*	[56]
Ovariectomized rat with periodontitis	120 mg/kg daily for 7 weeks	*Blautia*, *Allobaculum*, *norank_f_Bacteroidales_S24-7_group*, and *Roseburia* were increased; *Lactobacillus* was decreased.	[57]
HFD-induced obese rats	100 mg/kg orally once a day for 18 weeks	174 key OTUs were decreased, and 94 OTUs were enriched; *Allobaculum*, *Blautia*, *Bacteroides*, *Butyricimonas*, *Phascolarctobacterium*, *Prevotella*, *unclassified Porphyromonadaceae*, and *unclassified Ruminococcaceae* were enriched.	[58]
Experimental autoimmune uveitis mice induced by interphotoreceptor retinoid binding protein peptide 161–180	100 mg/kg daily for 14 days	Five genera were reduced including *Lactobacillus*; thirteen genera were increased including *Akkermansia* and *Oscillibacter.*	[59]
5% ethanol-induced alcoholic liver disease in C57BL/6J male mice	10, 50, and 100 mg/kg daily for 33 days	*Proteobacteria, Terrisporobacter,* and *Helicobacter* were increased; *Pseudoflavonifractor*, *Mucisirillum*, *Alistipes*, *Ruminiclostridium*, and *Lachnoclostridium* were decreased.	[60]
T2D rats induced by HFD	200 mg/kg/day for 6 weeks	*Bacteroidetes*, *Spirochaetaceae*, *Lactobacillaceae*, and *Peptostreptococcacea*e were increased; *Proteobacteria*, *Verrucomicrobi*, and *Enterobacteriaceae* were decreased.	[61]
Hepatitis rats induced by transplanting the stool of patients with diarrhea-predominant irritable bowel syndrome	200 mg/kg daily for 2 weeks	*Faecalibacterium*, *Ruminococcus*, *Clostridium* IV, *Gemmiger*, *Roseburia*, and *Clostridium* XI were reduced; *Clostridium* XIVa and *Bacteroides* were increased.	[62]
HFD-induced obese rats	150 mg/kg daily for 4 months	The species diversity and richness of gut microbiota were declined; *Bacteroidetes*/*Firmicutes* ratio was increased; *Bacteroidaceae*, *Rikenellaceae*, and *Bacteriodes* were enriched; *Coriobacteriia*, *Erysipelotrichi*, *Gammaproteobacteria*, *Christensenellaceae*, *Dehalobacteriaceae*, *Peptococcaceae*, *Dorea*, *Roseburia*, and *Blautia* were decreased.	[63]
HFD-induced obese rats	Oral 150 mg/kg daily for 6 weeks	The diversity and richness of gut microbiota was decreased. *Fusobacteria*, *Proteobacteria, Erysipelotrichaceae_incertae_sedis*, *Peptostreptococcaceae_incertae_sedis*, *Bacteroides*, *Escherichia-Shigella*, *Anaerostipes*, *Fusobacterium*, and *Phascolarctobacterium* were enciched; *Roseburia*, *Prevotella*, *Allobaculum*, *Faecalibacterium*, *Oscillibacter*, and *Desulfovibrio* were reduced.	[64]
HFD-induced obese rats	100 mg/kg daily for 8 weeks	The species diversity and richness of gut microbiota was declined; *Clostridium* XlVa, *Flavonifractor*, *Lachnospiracea_incertae_sedis*, *Roseburia*, and *Clostridium* XI were inhibited. *Butyricicoccus*, *Fusobacteria*, *Allobaculum*, *Parasutterella, Bacteroides*, *Blautia*, *Lactobacillus*, *Phascolarctobacterium*, and *Klebsiella* were enriched;	[65]
HFD-induced hyperlipidemia in male wistar rats	Oral 150 mg/kg daily for 4 weeks	*Prevotella*, *Clostridium*, *Sutterella*, and *Escherichia* were decreased; *Bacteroides*, *Blautia*, *and Parabacteroides* were increased.	[66]
5-fluorouracil-induced intestinal mucositis in rats	100 mg/kg daily for 8 days	*Proteobacteria* and *Verrucomicrobia* were decreased; *unclassified_f_Porphyromonadaceae*, *Firmicutes*, *Tenericutes*, *unclassified_f_Lachnospiraceae*, *Lactobacillus*, *Prevotella*, *unclassified_o_Clostridiales*, *Ruminococcus*, and *Clostridium* IV were enriched.	[67]
T2D mice induced by streptozotocin and HFD	100 mg/kg daily for 6 weeks	*Enterobacter* and *Enterococcus* were decreased; *Lactobacillus*, *Bifidobacterium*, and *Bacteroidetes* were increased.	[68]
T2D rats induced by HFD and high sucrose	500 mg/kg daily for 4 weeks	*Clostridia*, *Bacteroidetes*, *Prevotellaceae*, *Alloprevotella*, and *Lactobacillales*, were increased; *Lachnospiraceae*, *Bacteroidales*, *Desulfovibrio*, and *Rikenellaceae* were reduced	[69]
*Apc*^min/+^ mice feeding HFD	Blended into HFD at 500 mg/kg for 12 weeks	*Verrucomicrobia*, *Bacteroidetes*, *Akkermansia, Bacteroides, and Prevotellaceae_uncultured* were decreased; *Firmicutes, Lachnospiraceae_incertae_sedis, Bacteroidaceae, Bacteroides,* and *Bilophila* were enriched	[70]

BBR: berberine; DSS: dextran sodium sulfate; HFD: high-fat diet; NAFL: non-alcoholic fatty liver; T2D: type 2 diabetes.

In recent years, plenty of research using animal models of diseases has disclosed that BBR can ameliorate diseases by influencing the gut microbiota composition (Table 1). Among these studies, a high-fat diet (HFD)-induced disease model, dextran sodium sulfate (DSS)-induced disease model, and type 2 diabetes (T2D) model are the three most commonly used.

The disease model induced by HFD is commonly used to investigate the effect of BBR on the gut microbiota. In HFD feeding-induced insulin resistance, atherosclerosis, NAFL, and other diseases, the alleviation of disease and changes in the composition of gut microbiota were detected after BBR treatment. Liu et al. [44] studied the action of BBR on the gut microbiota of rats with HFD-mediated insulin resistance. The increase in fasting plasma glucose, fasting insulin, and homeostasis model assessment of insulin resistance (HOMA-IR) caused by HFD feeding decreased after BBR treatment; in addition, *Bifidobacterium* and *Lactobacillus* were enriched and *E*. *coli* was inhibited after BBR treatment. In HFD-induced atherosclerotic mice, the symptoms and the development of atherosclerosis in mice were improved after BBR treatment, and it was also observed that the diversity and richness of gut microbiota of mice decreased and the abundances of *Akkermansia* and *Bacteroides* increased [45]. In another study, BBR treatment could enrich *Firmicutes* and *Verrucomicrobia* and decrease *Bacteroidetes* and *Proteobacteria* in HFD-induced atherosclerotic mice [46]. In addition, in HFD-induced atherosclerotic mice, *Actinobacteria*, *Roseburia*, *Blautia*, *Allobaculum*, *Alistipes*, and *Turicibacter* were increased and *Proteobacteria* was reduced after treatment with BBR [47]. In HFD-induced NAFL rats, BBR reduced the weight of rats, total cholesterol, triglycerides, and low-density lipoprotein-cholesterol contents, and the improved phenotype was associated by an increase in *Bacteroidetes* and *Proteobacteria* and a decrease in *Firmicutes* and *Cyanobacteria* [48]. In another study, *F*. *prausnitzii* was reduced and the level of *Bacteroides* was elevated in HFD-induced NAFL rats after treatment with BBR [49].

DSS is a chemical agent widely used to induce colon disease. In these models, the gut microbiota composition plays a vital role in disease progression. However, BBR can reshape gut microbiota disorders and alleviate DSS-induced intestinal diseases. After treating DSS-induced ulcerative colitis with BBR, inflammation and intestinal barrier function were improved [50]. In addition, compared with the model group, the relative abundances of *Mucispirillum, Proteus, Oscillospira, and Allobaculum* decreased and the relative abundances of *Lactobacillus, Desulfovibrio, Ruminococcus, Parabacteroides, Sutterella, and Akkermansia* increased [50]. In another DSS-induced colitis model, the relative abundances of *Desulfovibrio, Proteobacteria, Streptococcaceae, Enterococcaceae*, *and Erysipelotrichale* decreased, while the relative abundances of *Eubacterium* and *Bacteroides* increased after BBR treatment [51]. In a colon cancer model induced by azoxymethane/DSS, BBR treatment improved the hypoplasia of the crypt and hyperplasia of adenoma in the mucosa and reduced the occurrence of colon cancer [52]. In addition, the relative abundances of *Actinobacteria*, *Verrucomicrobia*, *Bifidobacterium*, *Barnesiella*, and *Odoribacter* decreased and the relative abundances of *Alloprevotella*, *Flavonifractor*, *Oscillibacter*, and *Parabacteroides* increased after BBR treatment [52].

Several studies have used T2D animal models to investigate the relationships among BBR, diabetes, and gut microbiota composition, and the results show that BBR can relieve the symptoms of diabetes by regulating the gut microbiota composition. Using ob/ob mice as an experimental model, BBR treatment decreased blood glucose and lipids, and increased some intestinal bacteria such as *Nterobacter*, *Escherichia-Shigella, I*. *sedis*, *Akkermansia*, and *Bacteroides* [53]. In db/db mice with T2D, BBR could effectively regulate glucose metabolism and restore glucose homeostasis, and the abundance of *Saccharibacteria*, *Deferribacteres*, *Actinobacteria*, and *Firmicutes* was reduced [54]. In another study, BBR treatment reduced *Prevotella* and *Proteus* numbers and increased *Lactobacillus*, *Butyricimonas*, *Ruminococcus*, *Akkermansia*, and *Coprococcus* numbers [55].

In summary, BBR can not only change the composition of the gut microbiota in normal animals, but also alleviate HFD-induced insulin resistance, DSS-induced colitis, T2D, and other diseases by regulating the composition of the gut microbiota.

### 2.2 BBR regulates the metabolism of gut microbiota

**Fig. 1:**
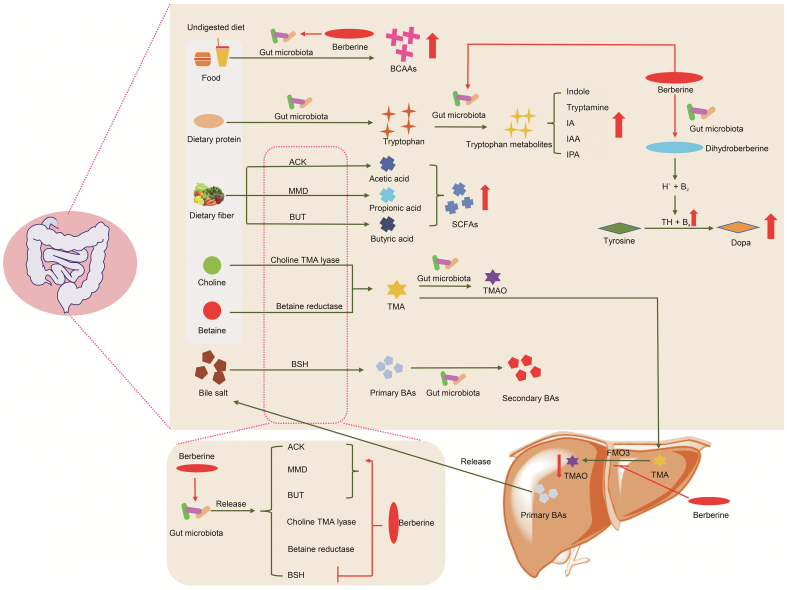
Typical gut microbiota metabolites and the influences of berberine (BBR) on production of these metabolites. ACK: acetate kinase; BAs: bile acids; BCAAs: branched-chain amino acids; BUT: butyryl-CoA:acetate-CoA transferase; BSH: bile salt hydrolase; B_2_: dihydrobiopterin; B_4_: tetrahydrobiopterin; FMO3: flavin-containing monooxygenase 3; IAA: indole-3-acetic acid; IA: indoleacrylic acid; IPA: indole-3-propionic acid; MMD: methylmalonyl-CoA decarboxylase; SCFAs: short-chain fatty acids; TMAO: trimethylamine *N*-oxide; TMA: trimethylamine.

**Fig. 2:**
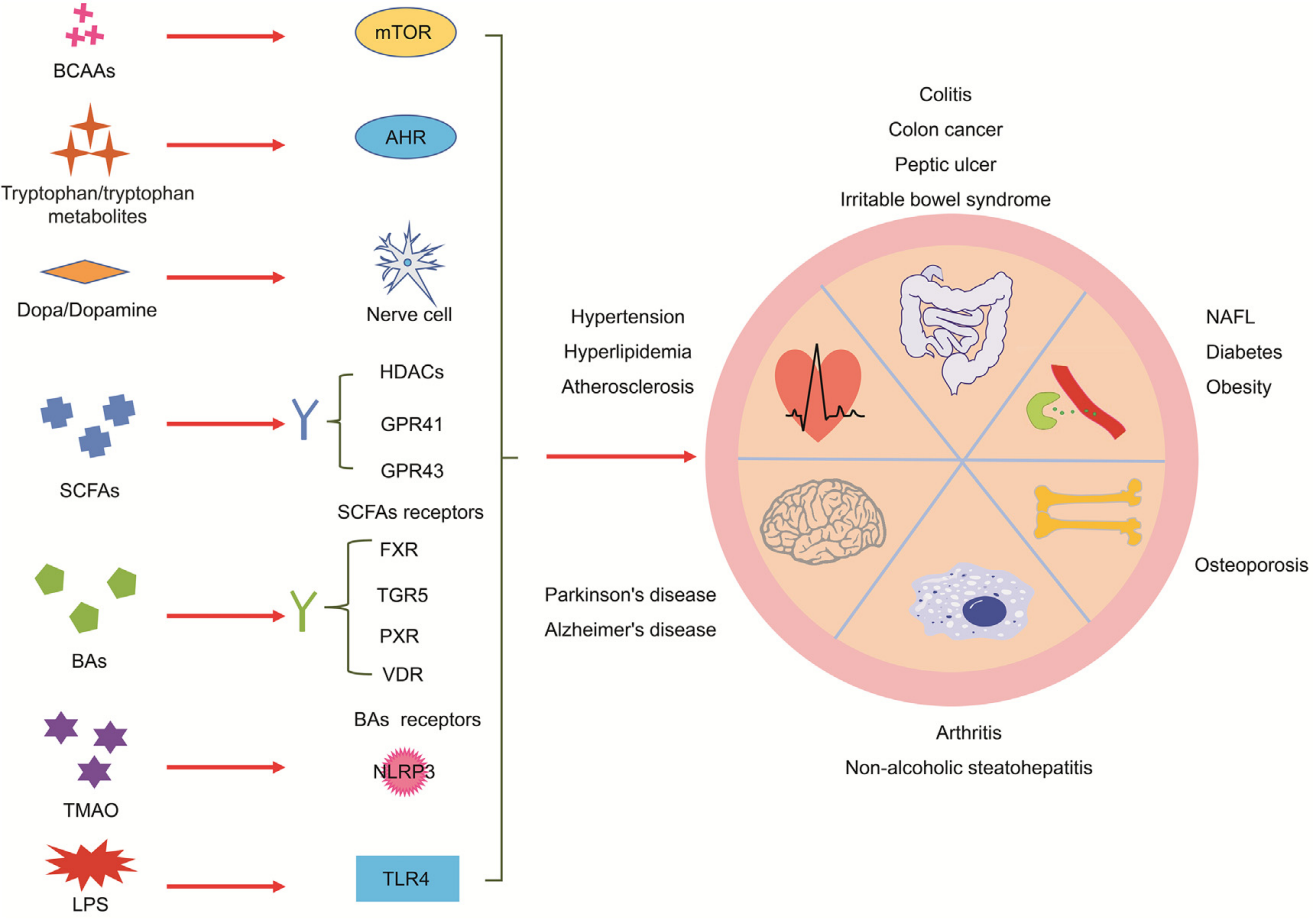
Targets of gut microbiota metabolites and the diseases related to these metabolites. AHR: aryl hydrocarbon receptor; FXR: farnesoid X receptor; GPR: G-protein-coupled receptor; HDACs: histone deacetylases; LPS: lipopolysaccharides; mTOR: mammalian target of rapamycin; NLRP3: nod-like receptor family pyrin domain containing 3; NAFL: non-alcoholic fatty liver; PXR: pregnane X receptor; TGR5: Takeda G protein-coupled receptor 5; TLR4: Toll-like receptor 4; VDR: vitamin D receptor.

Gut microbiota can generate and release a range of metabolites that include, but are not limited to, SCFAs, BCAAs, BAs, and TMA [[27], [28], [29]]. These metabolites have a great significance in cardiovascular [71,72], NAFL [73,74], Alzheimer's [75], and other diseases [76,77]. Therefore, regulating the metabolism of these metabolites has become necessary in treating diseases and maintaining normal physiological functions of the body [77]. Many studies have demonstrated that the efficacy of BBR on diseases is related to regulating gut microbiota metabolites by BBR (Fig. 1, Fig. 2).

#### 2.2.1 SCFAs

SCFAs are carboxylic acids with two to six carbon atoms [78]. The gut microbiota primarily synthesizes the SCFAs, mainly including butyric acid, acetic acid, and propionic acid, through the fermentation of undigested dietary fibers [79,80]. The SCFAs produced in the intestine can directly act on receptors on the intestinal wall, or be absorbed and then act on receptors in other parts of the body to produce pharmacological effects. There are mainly two types of SCFAs receptors: histone deacetylases (HDACs) and G-protein-coupled receptors (GPR41 and GPR43) [[81], [82], [83]]. HDACs play important roles in chromosome structure modification and gene expression regulation [84]. GPR41 and GPR43 have a regulatory effect on various systems of the body, such as the central nervous system, metabolic system, and cardiovascular system [[85], [86], [87]]. SCFAs can act on these receptors to regulate multiple sclerosis [88], atherosclerosis [89], cancer [90], colitis [91], diabetes [92], and other diseases (Fig. 2). Butyryl-CoA:acetate-CoA transferase (BUT), acetate kinase (ACK), and methylmalonyl-CoA decarboxylase (MMD) are the key enzymes for the synthesis of butyric acid, acetic acid, and propionic acid, respectively [93,94] (Fig. 1). Bacteria such as *Bacteroidetes*, *Firmicutes*, *Coprococcus*, *Fusobacteria*, *Actinobacteria*, *Roseburia*, *Allobaculum*, *Blautia*, *Butyricoccus*, *and Phascolarctobacterium* are related to the formation of SCFAs [95,96]. Therefore, regulating the activity and expression of the above enzymes and the composition of gut microbiota will change the content of SCFAs, subsequently regulating the physiological functions of the body.

BBR can regulate the content of SCFAs through two main mechanisms (Fig. 1). One is by modulating the expression and activity of the enzymes responsible for SCFA biosynthesis. Wang et al. [97] used BBR to cultivate the gut microbiota isolated from rats and found that acetic acid, butyric acid, and propionic acid contents increased after treatment with BBR. Additionally, the expression and activity of key enzymes involved in SCFA formation, such as ACK, MMD, and BUT, increased. BBR can improve intestinal hypoxia, restore the balance of intestinal energy supply, reduce the infiltration of inflammatory cells, reduce synovial hyperplasia, and downregulate the arthritis index and foot swelling degree. In addition, BBR promotes the production of butyric acid by increasing the activity of BUT [56]. BBR (10 and 20 μg/mL) can increase the expressions of BUT and two butyrate synthesis precursor substances (crotonyl-CoA and butyryl-CoA), subsequently lowering blood lipid and glucose levels [53]. These studies showed that BBR can regulate the production of SCFAs by improving the expressions and activity of the enzymes, thereby exerting downstream pharmacological effects.

Another mechanism by which BBR regulates the content of SCFAs is via regulating the abundance of SCFA-producing bacteria. BBR can effectively improve alveolar bone loss by inhibiting osteoclast activity, increasing osteoblasts, and improving bone metabolism in OVX rats with periodontitis; further, increased butyric acid concentration in feces was detected in BBR-administered rats. Furthermore, the relative abundances of *Blautia*, *Bacteroidales*, and *Roseburia* in the gut microbiota increased after BBR treatment [57]. In HFD-induced obese rats, BBR decreased the adiposity index and body weight. In addition, the fecal concentration of acetic acid and propionic acid, and the number of SCFA-producing bacteria such as *Allobaculum* and *Blautia* increased after BBR treatment [58].

#### 2.2.2 BAs

BAs are a class of molecules with a steroid structure that are generated from cholesterol in the liver [98]. After synthesis in the liver, primary BAs, mainly cholic acid (CA) and chenodeoxycholic acid (CDCA), combine with glycine or taurine to form bile salts and are secreted into the upper intestine along with cholesterol and coagulum. Some bile salts that reach the intestines are partially converted into secondary BAs, predominantly deoxycholic acid (DCA) and lithocholic acid (LCA) [99]. This conversion process involves the following two steps (Fig. 1). In the first step, bile salt hydrolases (BSH) specifically cut the amide bond of bile salts to dissociate primary BAs. In the second step, primary BAs are converted into secondary BAs under the action of the gut microbiota. For example, uncoupled CA and CDCA are catalyzed by microorganism 7α-dehydroxylase into LCA and DCA, respectively [[99], [100], [101]]. The expression of BSH has been reported in many gut bacteria such as *Lactobacillus*, *Bifidobacterium*, *Enterococcus*, and *Clostridium* spp. [99]. In addition, it was reported that the genera *Clostridium* and *Eubacterium* are the predominant intestinal species exhibiting BA 7α-dehydroxylating activity [102].

The primary BAs produced in the liver and secondary BAs produced in the intestines act on the BA receptors, which are highly expressed in the liver, intestine, brown adipose tissues, immune cells, and other tissues or cells, thereby regulating NAFL [103,104], liver cancer [105], diabetes [106], and other diseases. BA receptors predominantly comprise the Takeda G protein-coupled receptor 5 (TGR5) and the nuclear receptors, including vitamin D receptor, pregnane X receptor, and farnesoid X receptor (FXR) [[107], [108], [109]] (Fig. 2). Different BAs have different affinities with different BA receptors. For instance, DCA and LCA are the most efficient agonists for TGR5; however, CDCA has the highest affinity for FXR, followed by LCA, DCA, and CA. Therefore, the change in the BA pool regulates BA receptors and regulates the normal functioning of the body [107,108].

BBR exerts pharmacological effects by regulating the BA profile (Figs. 1 and 2). The serum free BA, total BA, and primary BA content increase after BBR treatment in mice, whereas the secondary BA content decreases; the effect is enhanced with an increase in BBR concentration [43]. In normal mice, BBR can inhibit the activity of BSH and reduce BSH-expressing bacteria, such as *Clostridium* spp. [29]. Meanwhile, taurocholic acid, tauroursodeoxycholic acid (TUDCA), and taurochenodeoxycholic acid (TCDCA) levels in the feces and liver and serum BA content are increased, and the cholesterol and free fatty acids are reduced [29]. Sun et al. [110] studied the influence of BBR on hepatic lipid metabolism of male mice. After treatment with BBR, the activity of BSH was inhibited, and the levels of some BAs such as CDCA, hyodeoxycholic acid, and DCA were reduced, while the levels of taurodeoxycholic acid, TCDCA, and TUDCA increased. Additionally, BAs can activate FXR to inhibit the expression of Cd36, which is a crucial protein allowing long-chain fatty acids to enter the liver and prevent obesity and triglyceride accumulation.

#### 2.2.3 TMAO

TMAO is produced by oxidation of TMA. Undigested choline, carnitine, and betaine in food (such as fish, eggs, offal, and beans) are converted into TMA under the action of enzymes produced by the gut microbiota [111] (Fig. 1). Bacteria related to TMA production include *Actinobacteria*, *Bacteroidetes*, *Firmicutes*, *Proteobacteria*, *Acinetobacter*, *Citrobacter*, *E*. *coli*, *Klebsiella*, *Proteus*, *Pseudomonas*, *Clostridium*, *Eubacterium*, *Sporomusa*, *Alcaligenes*, and *Escherichia* [112]. The microbial enzymes acting in the TMA synthesis process mainly include choline TMA lyase, betaine reductase, and carnitine oxidase/reductase [111,113]. Some of the TMA produced in the intestine is directly converted into TMAO in the intestines by oxidation [114], while some of the TMA is transferred to the liver and converted into TMAO under the action of flavin-containing monooxygenase 3 (FMO3) [114,115]. TMAO affects atherosclerosis [116,117], insulin resistance [118], cancer [119,120], and myocardial fibrosis [121,122]. The mechanism of action of TMAO is related to the activation of the nod-like receptor family pyrin domain containing 3 inflammasome [[123], [124], [125]] (Fig. 2).

Studies have revealed that BBR can influence TMAO, thereby exerting ameliorative effects on diseases (Fig. 1). After BBR treatment, the development of atherosclerosis is relieved in HFD-induced atherosclerosis mice. Further, the expression levels of matrix metalloproteinase-2, interleukin (IL)-6, and intercellular adhesion molecule 1 in aortic arch sections and the expression levels of tumor necrosis factor (TNF)-α, monocyte chemoattractant protein-1, IL-1β, and vascular cellular adhesion molecule-1 in the carotid artery are reduced. In addition, FMO3 and serum TMAO expressions decrease. These results indicate that BBR can relieve atherosclerosis, and this effect is correlated to the inhibition of the FMO3-TMAO pathway [46]. In another study, treatment with BBR alleviated atherosclerosis, reduced lipid levels, and decreased the concentration of inflammatory factors, including TNF-α, IL-1β, and IL-6. In addition, the abundance of enzymes (such as choline TMA lyase and betaine reductase) related to TMA production in mice was decreased. Therefore, BBR may regulate the enzymes involved in TMA synthesis to alleviate atherosclerosis [47].

#### 2.2.4 BCAAs

BCAAs (leucine, isoleucine, and valine) are three of nine essential amino acids that are not generated by our body; therefore, they must be obtained from the diet. The undigested part of foods such as dairy products, meat, fish, eggs, beans, nuts, and whole-grain products can be metabolized into BCAAs under the action of the gut microbiota [126,127] (Fig. 1). Many bacteria are involved in the biosynthesis of BCAAs, such as *Prevotella copri*, *Bacteroides vulgatus*, *C*. *clusters, the Bacillus-Lactobacillus-Streptococcus* group, *and Proteobacteria* (including *E*. *coli and Klebsiella* spp.) [[128], [129], [130]]. Mammalian target of rapamycin (mTOR) is a serine/threonine kinase that participates in regulating cell growth, proliferation, and development [131]. Abnormal activation of mTOR is correlated with various diseases, such as ischemic diseases and cancer [131,132]. BCAAs, especially leucine, are activators of the mTOR pathway and can regulate the physiological state of the body by activating mTOR [133]. For example, high levels of BCAAs contribute to activation of mTOR complex 1, which leads to insulin resistance through phosphorylation of insulin receptor substrate 1 [134]. In addition, free BCAAs are involved in protein synthesis, energy metabolism, and formation of the neurotransmitter glutamate [134,135].

BBR can regulate the biosynthesis of BCAAs to restore the function of the body, and the mechanism of regulation of BCAAs is related to inhibiting the microbial synthesis of BCAAs (Fig. 1). In obese mice, BBR can reduce body weight and the levels of serum total cholesterol, high-density lipoprotein, triglyceride, aspartate aminotransferase, and alanine aminotransferase. Furthermore, fasting serum glucose, insulin, and HOMA-IR index in insulin-resistant mice decreased after BBR treatment. Simultaneously, after BBR treatment, the relative abundances of BCAA-producing bacteria such as *Clostridium*, *Streptococcus*, *Clostridium*, *Proflagellate*, *Streptococcus*, and *Brevibacterium* were decreased, and the content of circulating BCAAs was reduced [30].

#### 2.2.5 Dopamine

Dopamine is a major neurotransmitter in the brain, and its levels in the brain are closely related to brain function [136]. The phenylalanine-tyrosine-dopa-dopamine pathway is the main dopamine synthesis pathway. The conversion of tyrosine into dopamine by tyrosine hydroxylase (TH) is the rate-limiting process of dopamine synthesis, and tetrahydrobiopterin acts as a coenzyme in this process [137]. Dopamine is mainly synthesized in brain neurons, but the metabolic pathway of phenylalanine-tyrosine-dopa-dopamine in microorganisms has also been confirmed [138]. Recent studies have pointed out that BBR can regulate the phenylalanine-tyrosine-dopa-dopamine metabolic process of the gut microbiota to promote the production of intestinal dopa/dopamine and transfer to the circulatory system, from which it can ultimately reach the brain and improve Parkinson's symptoms. Specifically, oral BBR supplies H^⋅^ through dihydroberberine (DhB) (reduced product of BBR by gut bacterial nitroreductase) and promotes the generation of tetrahydrobiopterin from dihydrobiopterin, which in turn enhances the activity of TH, thus increasing the generation of l-dopa by the gut microbiota [139] (Fig. 1).

#### 2.2.6 Tryptophan, indole, and indole derivatives

As an indispensable amino acid for the human body, tryptophan is primarily supplemented by the bio-transformation of dietary proteins. In the gastrointestinal tract, tryptophan metabolism follows three pathways: 1) the kynurenine pathway in both immune and epithelial cells, 2) the serotonin pathway in enterochromaffin cells, and 3) direct conversion by gut microbiota into metabolites including indole and indole derivatives such as indole-3-aldehyde, indole-3-acetic acid, indole-3-propionic acid, and indole-3-acetaldehyde [140]. Tryptophan and tryptophan microbial products, indole and indole derivatives, can activate the aryl hydrocarbon receptor (AHR) to regulate intestinal inflammation, gut hormone secretion, and gastrointestinal motility [141]. An article showed that BBR can significantly improve colitis in a DSS-induced colitis model [142]. This phenomenon occurs in a gut microbiota-dependent manner and is accompanied by an increase in fecal and serum tryptophan and indole derivatives, such as indole-3-propionic acid and indole-3-acetic acid. Furthermore, activation of AHR was observed in the colon tissue of the BBR group, and co-incubation of the Caco-2 cell monolayers and the culture supernatants of gut microbiota confirmed this result. This study demonstrated that BBR can improve colitis by promoting the microbial metabolism of tryptophan [142].

#### 2.2.7 Lipopolysaccharides

Lipopolysaccharide (LPS) is the main ingredient of the cell wall of gram-negative bacteria. Upon entering the circulatory system, LPS can stimulate Toll-like receptor 4 (TLR4) and lead to recruitment and activation of MyD88 adaptor and NF-κB transcription factor, resulting in the expressions of type I interferons, interferon (IFN)-inducible chemokines, and proinflammatory cytokines such as TNF and IL-1 [143]. Increased circulating LPS can promote inflammatory responses, insulin resistance, obesity, T2D mellitus, NAFL, chronic hepatitis, and other diseases [144,145]. Therefore, regulating the amount of plasma LPS is an important therapeutic approach to treating diseases such as NAFL. Recent studies have shown that BBR can regulate the concentration of plasma LPS by modulating gut microbiota. In HFD-induced obese rats, BBR treatment reduced the concentrations of plasma LPS and decreased the gut levels of gram-negative bacteria such as *E*. *coli*. In addition, the innate immune receptor TLR4 for LPS and the subsequent immune pathway (NF-κB pathway) were inhibited [44]. In addition, in db/db mice, treatment with BBR can reduce the levels of plasma LPS and gram-negative bacteria, including *Prevotella* and *Proteus*, and increase the levels of gram-positive bacteria (*Lactobacillus*). Meanwhile, the ameliorative effect of BBR on intestinal barrier inflammation is correlated with the downregulation of TLR/NF-κB signaling pathways [55]. The results demonstrate that BBR can regulate gut microbiota composition and plasma LPS concentration to improve diseases.

## 3 Direct metabolism of BBR by gut microbiota

**Fig. 3:**
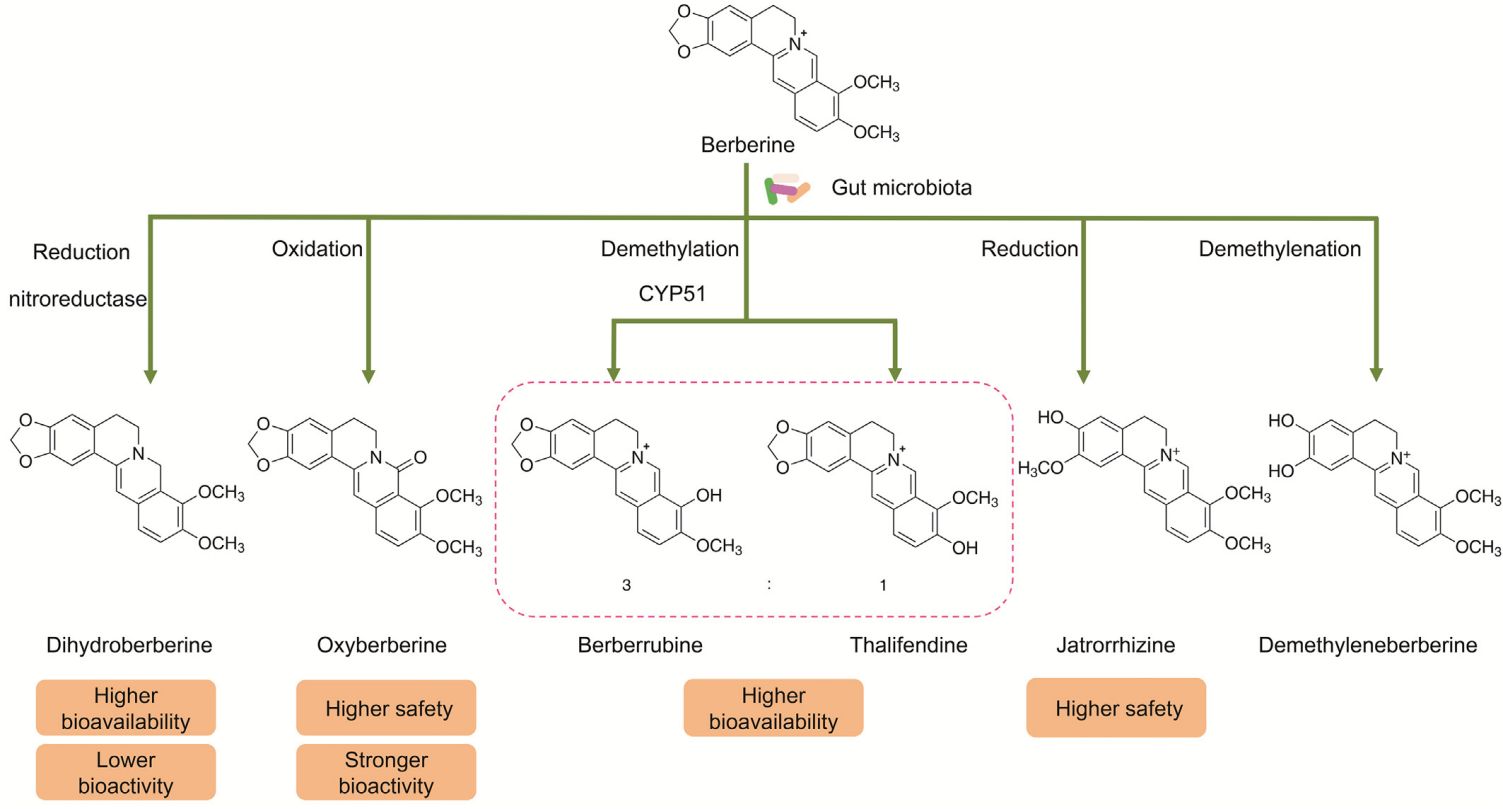
Chemical transformation of BBR under the action of gut microbiota.

With the rapid progress of metagenomics, studies have found that gut microbiota contains rich and diverse genes, in total representing several orders of magnitude more genes than the human genome [[146], [147], [148]]. The large number, different species, and complex and diverse genes endow the gut microbiota with powerful metabolic capabilities. Diets, drugs, antibiotics, and substances secreted by human cells can be bio-transformed by the gut microbiota into various secondary metabolites. The reactions provided by the gut microbiota include reduction, oxidation, demethylation, and isomerization [31]. The application of modern technologies shows that BBR can be transformed by the gut microbiota into DhB, berberrubine, demethyleneberberine (DMB), jatrorrhizine, and oxyberberine (OBB), as shown in Fig. 3. These metabolites have similar pharmacological effects to BBR, but compared with BBR, each has its own characteristics and advantages.

### 3.1 DhB

DhB, a reduction product of BBR, is produced under the catalytic activity of the gut microbiota [31]. Feng et al. [26] treated cultivated intestinal bacteria (*S*. *aureus*, *Enterococcus faecium*, *Enterococcus faecalis*, *Enterobacter cloacae*, *E*. *coli*, *Staphylococcus*, *Pseudomonas aeruginosa*, etc.) with BBR, and a decrease in BBR and DhB production was detected. Reports have pointed out that the intestinal microbial metabolite DhB transformed from BBR may be the key to explaining the intestinal absorption of BBR. Compared with BBR, DhB has a higher intestinal absorption rate (approximately five times that of BBR) and lower bioactivity [26,149]. The absorption of BBR in the form of DhB from the intestine into the circulatory system involves two processes: the conversion of BBR into DhB under the catalysis of gut microbiota enzymes and the conversion of DhB absorbed by intestinal epithelial cells into BBR through non-enzymatic oxidation reactions [26]. The conversion of BBR into DhB is carried out under the catalysis of gut microbial nitroreductase [150]. The intestinal microorganisms that produce nitroreductase include *Enterobacter*, *Escherichia*, *Shigella*, and *Bacteroides* [26,151]. The converted DhB can be absorbed by the intestinal epithelial tissue and then oxidized to BBR. The conversion rate of DhB into BBR in the intestinal epithelial tissue is approximately 80%; therefore, it is difficult to detect DhB in plasma [26]. In addition, studies have shown that the oxidation of DhB to BBR is a non-enzymatic reaction that is unrelated to cytochrome P450 (CYP450) and monoamine oxidase; however, some superoxide anions and metal ions seem to play an indispensable role in this process. Therefore, the activity and content of nitroreductase have become decisive factors that determine the speed of the BBR-DhB-BBR conversion process.

### 3.2 Berberrubine, thalifendine, and DMB

Demethylation is the main mechanism of microbial BBR metabolism. There are three main demethylation products of BBR: berberrubine, thalifendine, and DMB [31]. Berberrubine and thalifendine are products of the demethylation of the methoxy groups at the C-9 and C-10 positions of BBR, respectively, but DMB is a product of demethylation of the five-membered rings of the BBR dimethoxy subunit. Sterol 14α-demethylase (CYP51) belongs to the family of CYP450 enzymes and is present in many bacteria, lower eukaryotes, and mammals [152]. Berberrubine and thalifendine are both products of CYP51 (secreted by the gut microbiota) that catalyze BBR demethylation [153]. The bacteria in the gut microbiota, including *Enterococcus* *faecalis*, *Staphylococcus epidermidis*, *Enterobacter* *cloacae*, and *Enterococcus* *faecium,* contribute to the high yield of berberrubine [153]. In addition, cultivation of different concentrations of BBR with the same intestinal contents in vitro found that although the content of BBR increased, the percentage of BBR metabolism decreased, suggesting that the concentrations of gut bacteria and activities of CYP51 in each cultivation system were constant. Therefore, changing the number of gut bacteria and the content and activity of CYP51 may regulate the conversion of BBR into berberrubine or thalifendine, thereby affecting the pharmacological activity of oral BBR.

Research has shown that these three metabolites of BBR have similar biological activities as BBR; for example, BBR and berberrubine can alleviate intestinal inflammation [154,155], and BBR and DMB can alleviate liver diseases [156,157]. However, the content of the three BBR demethylation products was different after oral administration of BBR. Some scholars have pointed out that the primary metabolites of BBR in the small intestines are DMB glucuronide and berberrubine, and the levels of DMB and berberrubine with much higher levels than that of BBR are detected in plasma [158]. In addition, berberrubine shows better fat solubility and higher absolute bioavailability (31.6%) than BBR (<1%) [10,11,158,159]. In summary, it can be seen that the demethylated metabolites of BBR have more advantages than BBR and may be the basis for the pharmacological activity of BBR.

### 3.3 Jatrorrhizine

The five-membered dioxymethylene ring in BBR can be cleaved under the action of the gut microbiota to produce jatrorrhizine [31], but it is unclear what microorganisms drive this conversion process. Jatrorrhizine has a higher safety than BBR as a microbial reduction product of BBR. The LD_50_ of jatrorrhizine in mice is approximately 5500 mg/kg, while the LD_50_ of BBR is 763 mg/kg [160]. Studies have shown that both jatrorrhizine and BBR have pharmacodynamic action against hypercholesterolemia and rectal cancer [[160], [161], [162], [163]]. In addition, jatrorrhizine has been recommended as a gastric motility agent because of its effect in promoting gastric motility [164].

### 3.4 OBB

As a compound that naturally occurs in a variety of plants such as *Coptis chinensis* Franch [165], OBB is also an oxidation product of BBR after transformation by gut microbiota. Structurally, OBB is the product of oxidation of the C-8 position of BBR to hydroxyl [31]. Functionally, OBB and BBR have similar bioactivities, such as anti-inflammatory, anti-diabetic, and anti-colitis effects. However, studies have demonstrated that at the same dose, OBB is more effective in relieving inflammation and diabetes than BBR [166,167]. In addition, OBB is safer than BBR in mice; the *LD*_*50*_ of OBB (5243.6 mg/kg) is far greater than that of BBR (approximately 713.57 mg/kg) [168]. Therefore, OBB may be a material base for the pharmacological activity of BBR in vivo. The conversion of BBR into OBB is driven by gut microbiota, and the conversion rate can reach 12.42% in normal SD rats. It has been found that six bacteria in the gut microbiota, namely, *Bifidobacterium longum*, *Lactobacillus acidophilus*, *Streptococcus faecalis*, *E*. *coli*, *Pseudomonas aeruginosa*, and *S*. *aureus*, can convert BBR into OBB, and *Streptococcus* *faecalis* is the most productive [166].

## 4 Other complex interactions

In addition to the direct effects that can produce metabolites acting directly on targets of diseases, there are complex interactions between BBR and gut microbiota. These complex interactions involve tissues and proteins associated with disease development and drug transportation. Typical tissues and proteins include the intestinal barrier and P-glycoprotein.

### 4.1 Intestinal barrier

**Fig. 4:**
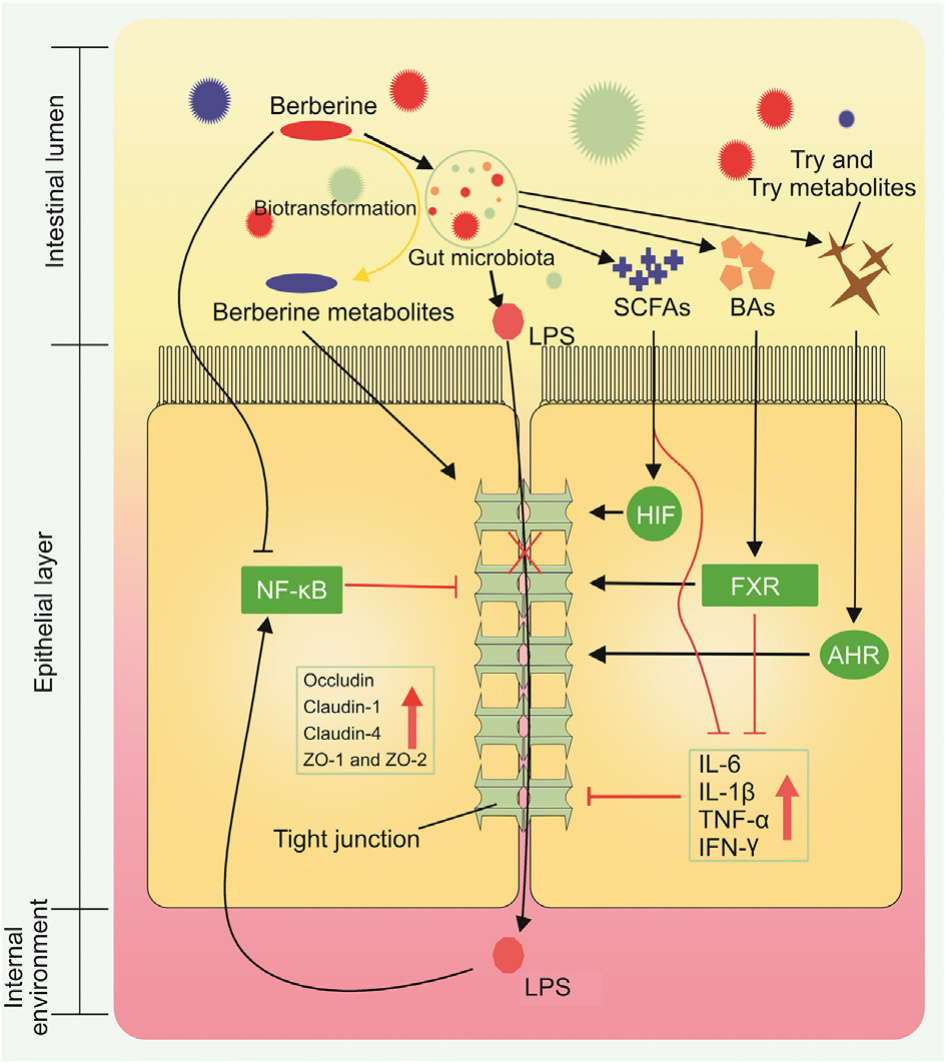
BBR regulates the intestinal barrier through the gut microbiota. IL: interleukin; HIF: hypoxia inducible factor; TNF-α: tumor necrosis factor-α; Try: tryptophan; ZO: zonula occludens.

The intestinal barrier is a layer of physical and functional barriers between the gut lumen and immunocompetent submucosa [169]. The intestinal barrier comprises four distinct functional layers: 1) epithelial cells, which serve as a ‘functional barrier’ by releasing alkaline phosphatase to detoxify bacterial endotoxins (LPS), and pathogen-associated molecular patterns; 2) the mucus layer, which acts as a ‘physical barrier’ to prevent direct contact between gut microbiota and intestinal epithelia; 3) tight junctions between epithelial cells, which prevent the transport of LPS and other compounds derived from gut microbiota; and 4) specialized functional cells, such as Paneth cells, which secrete antimicrobial compounds [[169], [170], [171], [172]]. The functions of the intestinal barrier are directly correlated with a number of diseases, including metabolic diseases [173], schizophrenia [174], autism [174], irritable bowel syndrome [175], inflammatory bowel disease [176], coronary heart disease [177], chronic kidney disease [178], chronic pancreatitis [179], and various cancers [180]. In view of the significance of the intestinal barrier in the homeostasis of our body function, some scholars have conducted research on how to alleviate damage to the intestinal barrier. Among these studies, BBR has been revealed as a representative of phytotherapy (Fig. 4).

#### 4.1.1 BBR regulates gut microbiota to improve intestinal barrier damage

The gut microbiota and intestinal barrier are physically and functionally related as a pair of systems that live next to each other. Studies have revealed that gut microbiota has the capacity to regulate the function of intestinal barrier by regulating intestinal epithelial cell renewal, influencing intestinal permeability, and modulating antimicrobial protein expression and the mucus layer [181]. Loss of function in the intestinal barrier can contribute to a series of diseases, such as irritable bowel syndrome and inflammatory bowel disease. Improvement of the intestinal barrier function by restoring the composition of gut microbiota is now considered as an important approach to treating diseases [176,177]. In rats with NAFL induced by HFD, administering 150 mg/kg BBR relieved the symptoms of fatty liver; additionally, the intestinal mucosal villi were plump and arranged tightly and neatly compared to the model group. In addition, the richness and diversity of gut microbiota in the BBR group decreased and abundances of *Bacteroidetes* and *Proteobacteria* increased significantly, and those of *Firmicutes* and *Cyanobacteria* decreased significantly compared with those in the control group [48]. The results indicated that restoration of the intestinal barrier and gut microbiota is associated with efficacy of BBR.

Among a large number of gut bacteria species, *Akkermansia* has attracted the interest of more and more scholars due to its close relationship with health in animals and humans. *Akkermansia*, a type of bacteria, settles on the mucus layer and uses mucin as a source of nutrition [45]. Researches have disclosed that the relative abundance of *Akkermansia* is negatively correlated with intestinal inflammatory and metabolic diseases. In addition, *Akkermansia* supplementation can increase intestinal epithelial tight junction protein levels and improve the intestinal barrier [182]. Therefore, modulation of *Akkermansia* has attracted considerable attention for the treatment of diseases such as ulcerative colitis [183]. In HFD-induced atherosclerosis in *ApoE*^-/-^ mice, BBR significantly reduced atherosclerosis in mice and increased the level of *Akkermansia* [45]. In addition, BBR significantly decreased HFD-induced metabolic LPS, lowered arterial and intestinal inflammatory cytokines and chemokines, increased intestinal tight junction proteins (zona occludens 1 (ZO-1) and occludin), and increased the thickness of the colonic mucus layer [45]. These results suggest that modulation of *Akkermansia* ameliorates atherosclerosis in mice. However, additional studies, such as fecal transplantation of *Akkermansia*, are required to confirm these results.

#### 4.1.2 BBR regulates gut microbiota metabolites to improve intestinal barrier damage

SCFAs, especially butyric acid, can not only act as the main nutritional source of colon cells, but also stabilize hypoxia inducible factor (HIF) levels and promote tight junctions in intestinal epithelial cells to maintain the stability of the intestinal barrier [[184], [185], [186]]. The destruction of tight junctions between intestinal epithelial cells will help intestinal hazardous materials, such as LPS, enter the systemic circulation, and then lead to activation of the TLR/NF-κB signaling pathway and promote the release of pro-inflammatory cytokines [169]. In db/db mice, intragastric administration of BBR 136.5 mg/kg daily for 11 weeks increased the expressions of tight junction proteins (occludin and ZO-1) and alleviated the gap between intestinal epithelial cells. In addition, the content of serum LPS was reduced, the SCFA content in the feces increased, and the number of SCFA-producing bacteria (including *Butyricimonas*, *Coprococcus*, *and Ruminococcus*) increased [55]. The study indicated that BBR can regulate the content of SCFAs and LPS to ameliorate intestinal barrier damage. In another study, treatment with BBR can relieve collagen-induced arthritis, downregulate the diversity and richness of gut microbiota, and elevate the level of butyrate-producing bacteria. In addition, the level of the intestinal butyrate, the activity of BUT, and the intestinal expression of HIF-1α increased after BBR treatment*.* Furthermore, co-administration of a BUT inhibitor diminished the anti-arthritic effect of BBR [56]. These results indicate that BBR can regulate the intestinal content of SCFAs to improve the intestinal barrier and ameliorate diseases.

Other types of gut microbiota metabolites associated with the regulatory actions of BBR on the intestinal barrier are microbial BAs and tryptophan catabolites. BAs play important roles in protecting the function of the intestinal barrier by modulating the survival and death of epithelial cells, permeability of tight junctions, secretion of mucus layer, and epithelial secretion of cytokines [[109], [187]]. BA-induced activation of FXR and TGR5 can restrain the production of inflammatory cytokines such as TNF-α and IFN-γ, which is helpful for reducing inflammation and epithelial cell penetration. After oral administration, BBR can modulate lipid metabolism by regulating microbial BA metabolism and the activation of intestinal FXR [110]. Microbial tryptophan catabolites can control intestinal epithelial permeability by acting as AHR ligands that inhibit the activation of actin regulatory proteins [188]. After oral administration, BBR can improve intestinal barrier function by triggering AHR activation by microbial tryptophan catabolites [143].

#### 4.1.3 Modulatory effects of metabolites of BBR on the intestinal barrier

In addition to gut microbiota and gut microbiota metabolites, the microbial metabolites of BBR, including berberrubine, DMB, and OBB, can also improve intestinal barrier function. OBB can improve intestinal barrier function, as evidenced by the increased length of the mouse colon, reduced inflammatory cell infiltration and intestinal tissue-related inflammatory factors (IL-6, IL-10, IL-1β, IL-17, TNF-α, and IFN-γ), and increased level of tight junction proteins (ZO-1, ZO-2, occludin, and claudin-1). In addition, the pharmacological effect of OBB was stronger than that of BBR [166]. Berberrubine can increase the level of tight junction proteins (ZO-1, ZO-2, claudin-1, and occludin) and mucins (mucin-1 and mucin-2), alleviate inflammatory cell infiltration, and reduce intestinal tissue inflammatory factors (TNF-α, IL-1β, IL-10, IL-6, IL-4, and IFN-γ) [154]. DMB can activate the NF-*κ*B pathway to improve intestinal barrier function, and its effect shows dose-dependent tolerance [189].

### 4.2 P-glycoprotein

P-glycoprotein is an ATP-driven efflux pump, which is widely distributed in the large intestines, small intestines, hepatobiliary duct, and epithelial cells of the proximal tubule of the kidney. It plays an indispensable role in preventing the absorption of foreign materials into the intestine [[190], [191], [192]]. In the intestine, P-glycoprotein-mediated drug efflux is an important reason for the low bioavailability of BBR. Studies have shown that SCFAs can inhibit the activity of P-glycoprotein, and butyric acid has the strongest inhibitory effect, followed by propionic acid and acetic acid [193,194]. BAs downregulate the gene encoding P-glycoprotein and decrease the expression of P-glycoprotein [195]. Therefore, gut microbiota may regulate the absorption of BBR via the regulation of P-glycoprotein.

Previous studies have shown that P-glycoprotein inhibitors can improve the absorption of BBR and enhance its pharmacological effects. Human immunodeficiency virus protease inhibitors can inhibit intestinal P-glycoprotein activity and compete with BBR for P-glycoprotein, thus improving the intestinal absorption rate of BBR [196]. In addition, studies have shown that verapamil can increase the neuroprotective effect of BBR in rats with sporadic dementia and Alzheimer's disease induced by streptozotocin, and the mechanism is related to the inhibition of P-glycoprotein activity [197]. These results suggest that increased production of SCFAs and changes in BA profiles induced by BBR can further regulate the expression and activity of P-glycoprotein, and the intestinal absorption and pharmacological effects of BBR can eventually be altered.

## 5 Side effects of BBR and personalized use of BBR

The normal gut microbiota composition and metabolism are important for the maintenance of health [198]. When the ecological balance of the gut microbiota is disturbed, a series of diseases may be induced [199]. Although gut microbiota regulators can ameliorate disease by adjusting the composition and metabolism of the gut microbiota, unreasonable use of these regulators can also cause gut microbiota disorders and undesirable side effects [200]. BBR is a classic example of this sort of gut microbiota regulator. In the clinic, the main side effect of unreasonable application of BBR is diarrhea, which is similar to the side effects of antibiotics [201]. In patients with T2D, 500 mg of BBR three times a day for 13 weeks could cause multiple adverse abdominal reactions, including diarrhea, constipation, and abdominal pain [202]. In another study, BBR (200 mg/kg) administration for three weeks caused diarrhea in 62.5% of rats. In addition, changes in the metabolism and composition of gut microbiota in diarrheal rats were also detected. For example, the relative abundances of *Parabacteroides*, *Prevotellaceae_UCG-001*, and *Prevotellaceae_NK3B31_group* increased, the relative abundance of *Christensenellaceae_R-7_group* decreased, and cecal SCFA levels (acetic acid and propionic acid) were reduced. These results suggest that BBR may cause disorders in the gut microbiota and diarrhea [203]. In addition, disorders of BA metabolism can also cause diarrhea. For example, excessive BA synthesis and secretion or decreased absorption in the ileum can cause diarrhea [204]. It has been reported that BBR can cause an increase in serum BA, total BA, and primary BA content [43]. Disorders of BA metabolism may also be the mechanism by which BBR causes diarrhea; however, this mechanism needs to be further confirmed.

The composition of gut microbiota varies greatly among different persons, and this variation can lead to variations in drug toxicity and efficacy [205]. Therefore, personalized use of gut microbiota has emerged as an important way to enhance drug efficacy and reduce side effects [206,207]. Because of the common side effects, such as diarrhea and abdominal discomfort, it is necessary to use BBR personally in the clinic. Wang et al. [208] screened biomarkers for BBR use by exploring the differences in bioavailability of BBR between normal hamsters and hyperlipidemia hamsters (induced by HFD). Bioavailability of BBR in the HFD-fed hamsters, which showed higher intestinal nitroreductase activity, was higher than that in hamsters fed with normal diet. In addition, correlation analysis of clinical samples revealed a positive relationship between the activity of fecal nitroreductase and level of blood BBR. Therefore, fecal nitroreductase activity may serve as an important physiological indicator for personalized treatment of hyperlipidemia when using BBR.

## 6 Perspectives

### 6.1 The need for detection of the key gut bacteria and enzymes

Even though great achievements have been made regarding the interactions between gut microbiota and BBR, in most studies, only the associative relationships between gut microbiota species and metabolites of gut microbiota and BBR have been investigated. Specifically, the number of gut bacteria may change after BBR administration; however, not all of these bacteria might be responsible for the transformation of BBR and the production of gut microbiota metabolites such as SCFAs. To establish a causal relationship between gut microbiota and metabolites, animal models such as gnotobiotic and germ-free animals, and a variety of other methods, including in vitro and ex vivo incubation, antibiotic supplementation, and fecal microbiota transplantation should be employed. In addition to gut bacteria, the enzymes in charge of the transformation of BBR and synthesis of gut microbiota metabolites, such as BCAAs, need to be identified. In this aspect, Wang et al. [139] offered excellent examples, in which BBR ameliorates Parkinson's disease, as we have discussed in Section 2.2.5. However, the success of examples offered by Wang et al. [139] only explains a small proportion of the therapeutic effects of BBR, considering the versatility of BBR in treating diseases, and an increasing number of studies are needed to delve into the key gut bacteria and enzymes regulated by BBR when BBR is used for the treatment of other diseases such as colitis.

### 6.2 Separating and integrating the contributions of BBR, BBR transformed metabolites, and gut microbiota metabolites to therapeutic effects

After oral administration, three types of compounds may be absorbed: gut microbiota metabolites (such as SCFAs), BBR transformed metabolites, and BBR. All of these compounds can act on target organs, cells, and proteins to alleviate diseases such as obesity [209,210]. To predict the outcome of BBR, it is necessary to separate and integrate the contribution of each type of compound to the therapeutic effects of orally administered BBR. Nevertheless, gut microbiota and hosts are materially interlaced, and separating the contribution of each type of compound to the final therapeutic outcomes is rather challenging, considering that the gut microbiota and liver can transform BBR to the same metabolites. For example, BBR can be transformed into berberrubine, thalifendine, DMB, and jatrorrhizine in the liver [31], which are also the products of BBR when transformed by gut microbiota. To separate the contributions of the gut microbiota and liver to the production of BBR metabolites, development of new algorithms and use of gnotobiotic and germ-free animal models are necessary. Recently, Zimmermann et al. [211] offered an excellent example by separating the contribution of gut microbiota and host to the metabolism of brivudine in mice. Once the contribution of gut microbiota and hosts to the metabolism of BBR is separated, the establishment of a method to predict the therapeutic outcome of oral BBR by integrating each type of BBR metabolite to the therapeutic effects is another aim that needs to be achieved. Although no study has currently achieved this aim, we anticipate that this goal may be attained in the future with the establishment of new algorithms, animal models, organ models, and organoid models.

## 7 Conclusion

In recent years, gut microbiota has become a hotspot because of its role in disease development. The gut microbiota can interact with drugs, including BBR, thereby modulating their efficacy. There are two major methods by which gut microbiota and BBR directly interact with each other: BBR regulates the gut microbiota, and gut microbiota transforms BBR. Gut microbiota metabolites, such as tryptophan, and transformed BBR products, such as OBB, can directly act as targets to improve diseases. In addition to these two direct interactions, BBR may modulate the function of the intestinal barrier and activity of P-glycoprotein, two targets that can indirectly influence the development of diseases and directly influence the transportation of BBR through the body. Understanding the interactions between BBR and gut microbiota can not only explain the contradiction between the low bioavailability and conspicuous pharmacological activity of BBR, but also pave the way for further research on BBR based on gut microbiota and provide reference for clinical rational use of BBR in the treatment of diseases.

## CRediT author statement

**Hao Cheng:** Writing - Original draft preparation; **Juan Liu** and **Yuzhu Tan:** Writing - Reviewing and Editing; **Wuwen Feng:** Supervision, Writing - Reviewing and Editing; **Cheng Peng:** Conceptualization.

## Declaration of competing interest

The authors declare that there are no conflicts of interest.
